# Prediction of viral microRNA precursors based on human microRNA precursor sequence and structural features

**DOI:** 10.1186/1743-422X-6-129

**Published:** 2009-08-20

**Authors:** Shiva Kumar, Faraz A Ansari, Vinod Scaria

**Affiliations:** 1 GN Ramachandran Knowledge Center for Genome Informatics, Institute of Genomics and Integrative Biology, CSIR, Mall Road, Delhi 110 007, India

## Abstract

MicroRNAs (small ~22 nucleotide long non-coding endogenous RNAs) have recently attracted immense attention as critical regulators of gene expression in multi-cellular eukaryotes, especially in humans. Recent studies have proved that viruses also express microRNAs, which are thought to contribute to the intricate mechanisms of host-pathogen interactions. Computational predictions have greatly accelerated the discovery of microRNAs. However, most of these widely used tools are dependent on structural features and sequence conservation which limits their use in discovering novel virus expressed microRNAs and non-conserved eukaryotic microRNAs. In this work an efficient prediction method is developed based on the hypothesis that sequence and structure features which discriminate between host microRNA precursor hairpins and pseudo microRNAs are shared by viral microRNA as they depend on host machinery for the processing of microRNA precursors. The proposed method has been found to be more efficient than recently reported *ab-initio *methods for predicting viral microRNAs and microRNAs expressed by mammals.

## Background

MicroRNAs are a class of small non-coding RNAs which have recently attracted widespread attention due to their critical role in a wide spectrum of biological processes in multi-cellular eukaryotes. In animals, these small RNAs are processed from hairpin-forming precursors and exported to the cytoplasm where they are further processed and incorporated into a protein complex, the RNA Induced Silencing Complex (RISC) as a ~22 nucleotide long mature miRNA. This RNA-protein complex then affects regulation of gene expression by binding to the 3'UTR of messenger RNA and thereby causing a translational block [1]. Recent studies have shown that microRNA mediated gene regulation is widespread in eukaryotes and is presently known to encompass a wide spectrum of biological processes ranging from growth and differentiation to oncogenesis [2,3]. The entire set of biological processes in which microRNAs play a critical role is yet to be unraveled.

Recently viruses have also been shown to express microRNAs [4,5]. These virus-expressed microRNAs have been shown to not only regulate viral transcripts, but also host transcripts. Furthermore, recent evidence suggest that virus expressed microRNAs can play significant roles in the pathophysiology of HIV infection, including latency of the virus [6,7]. Moreover a microRNA residing in the *nef *gene has been shown to target its own gene formation in an auto regulatory loop [8,9] which is thought to be critical in long-term non-progression of HIV infection. Recent studies have also shown that Herpes Simplex Type-1 (HSV-1) expressed microRNA [10,11], derived from a latency associated transcript (LAT) can target critical host genes vital in mediating latency of disease.

Furthermore, microRNAs expressed by other pathogenic viruses could also regulate host transcripts and thus play important roles in the pathogenesis of disease. Thus it is imperative to understand the entire repertoire of virus expressed microRNAs in order to unravel the mechanisms of pathogenesis of diseases caused by viruses and for designing novel therapeutic strategies against the virus [12].

Computational methods [13] have been critical in the discovery of novel microRNAs many of which have been validated experimentally. For example, *miRseeker*, was used in the discovery of Drosophila expressed microRNAs [14] and *miRscan *facilitated the discovery of C elegans microRNAs [15]. These methods rely heavily on hairpin structures and the evolutionary conservation of sequence. Also, a phylogenetic shadowing [16] approach has been used for the discovery of novel human microRNAs. Furthermore, methods have been recently proposed that do not rely on conservation of microRNA sequences and have helped in discovery of human, mouse and rat microRNAs [17-19] as well as microRNAs in viruses [20,4]. While the former method takes into consideration thermodynamically stable RNA hairpins, the latter uses machine learning approach to predict microRNAs. However, note that virus expressed microRNA genes show rapid evolution. For example, herpes-virus expressed microRNAs do not share homologies with microRNAs expressed by other unrelated viruses or with that of the host, [21] but share homologies within closely related viruses [22] This necessitates the creation of newer and better algorithms for *ab-initio *prediction of microRNAs.

Viruses are dependent on the cellular biosynthetic machinery for the processing of microRNAs. Thus we hypothesize that sequence-structure features that differentiate true microRNA precursors from pseudo microRNA hairpins are critical for processing microRNAs. These features would be shared between host and viral microRNA precursors and thus help in the prediction of viral microRNAs. Moreover the microRNA processing mechanism is conserved across eukaryotes. This implies that sequence and structure features inherently unique or discriminative of microRNA precursors are also likely to be shared across eukaryotes.

We hypothesize that our method can therefore predict microRNAs in other eukaryotic organisms as well as discover novel non-conserved microRNAs systematically missed by prediction methods which rely on evolutionary conservation of sequences.

This proposed method employs Support Vector Machine trained on sequence-structure feature elements for an efficient discrimination between microRNA precursor hairpins and pseudo microRNA hairpins. We have validated this approach for a number of known viral as well as Chimpanzee, Mouse and Worm microRNA precursors derived from public databases. We see that proposed method is more accurate than the recently reported *ab-initio *microRNA prediction algorithms [18,23,24]. For viruses and mammals (as evidenced by prediction accuracies in *Pan troglodytes *and *Mus musculus*). The method does not perform better than other tools for *Caenorhabditis elegans *probably due to the difference in the base composition in the genome.

In fact, a genome-wide analysis has been done on Herpes Simplex virus (HSV-1) and furthermore a recent independent study has experimentally validated a subset of the prediction [10,11]. These provide evidence about the genome-wide applicability and reliability of our method for *ab-initio *prediction of microRNA precursors.

The method is fast enough for genome-wide predictions even in larger genomes and can aid in the discovery of both novel non-conserved microRNAs and novel virus-expressed microRNAs.

## Results

### Model creation and selection

**Figure 1:**
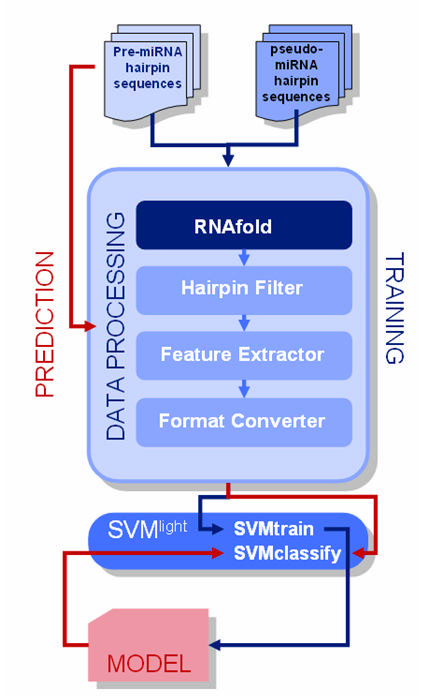
**Illustrative summary of the process flow in the presented method for microRNA precursor prediction**. The lines in red denote the process flow in prediction while the lines in dark blue denote the process flow during training.



The models were created by taking a non overlapping random samples amounting to half of the positive and negative datasets for training and were evaluated on the remaining dataset. The process of model generation and testing is summarized in Fig 1. One hundred such models were created and evaluated by random sampling. Models were analysed for sensitivity and specificity, where:

and



The model which had maximum specificity was selected for further evaluation and comparison with other existing tools. We selected model with maximum specificity rather than sensitivity to cut down on false positive predictions that may arise from cross-species predictions. The performance of the best models is summarized in Table 1. The model 031 was selected as it had better specificity than the other models and has a good sensitivity of 68%.

**Table 1:** Sensitivity and specificity measures of top 5 models with maximum specificity

**Sl No**	**Model ID**	**Sensitivity**	**Specificity**
1	MODEL 031	68%	87%

2	MODEL 034	69.7	85.32

3	MODEL 076	69.3	86

4	MODEL 001	77%	78%

5	MODEL 100	67%	78%

### Prediction of known virus precursors

MicroRNA precursor sequences were downloaded from miRbase for three classes of human viruses with experimentally verified microRNAs, which include Epstein Barr virus (EBV), Cytomegalovirus (CMV) and Kaposi Sarcoma Herpes virus (KSHV). They had 23, 11 and 13 entries respectively. The model correctly predicted 22 out of the 23, 11 out of 13 and 10/11 of the microRNAs expressed by EBV, KSHV and CMV respectively. The comparisons of different programs are summarized in Table 2.

**Table 2:** Comparison of the number of viral microRNAs predicted by the 3 *ab-initio *prediction algorithms-BayesmiRNAfind, mir-abela, mirSVM and our method on a dataset of viral microRNA precursors derived from mirBase

**Virus expressed microRNAs derived from miRbase**	**PREDICTIONS**			
**Virus**	**Bayes miRNAfind**	**Mir-abela**	**tripletSVM**	**MODEL 031**

Epstein-Barr Virus **(23)**	21 **-91.3**	18 **-78.26**	21 **-91.3**	22 **-95.65**

Cytomegalovirus **(11)**	10 **-90.91**	5 **-45.45**	7 **-63.63**	10 **-90.91**

Kaposi Sarcoma Herpesvirus (13)*	9 **-69.23**	5 **-38.46**	8 **-61.54**	11 **-84.62**

Herpes Simplex Virus (2) **(sequences were derived from literature)	2 **100%**	0 **0%**	0 **0%**	2 **100%**

The numbers denote the total number of positive predictions and numbers in brackets following the organism name denotes the total number of microRNA hairpins in mirRbase and that following the positive predictions denote the percentage of total predicted. (*)KSHV expresses only 12 microRNA hairpins. The 13^th ^sequence is one with a possible single nucleotide editing that was cloned. (**) For HSV, the sequences were derived from respective literature as the version of miRbase did not yet include the sequences.

### Comparison with other prediction programs

The dataset of microRNAs expressed by viruses was tested on three newer *ab-initio *microRNA prediction methods reported in literature, BayesmiRNAfind [24], TripletSVM [23] and mir-abela [18]. We find that our method performs better than the other methods.

### Testing on new datasets of Herpes Simplex virus expressed microRNAs

While this manuscript was in preparation, new microRNA sequences have been reported for Herpes Simplex virus (HSV) latency associated transcript (LAT) [10,11]. The two hairpin sequences that were experimentally validated were correctly predicted by our program as potential microRNA precursors.

The results of the prediction are summarized in Table 2.

### Comparison of the method on Eukaryotic microRNA precursors

To validate the models, we compared our method with a recently published method employing SVM on a set of microRNA precursor sequences derived from miRbase for *Pan troglodytes *(Chimpanzee), *Mus musculus *(Mouse) and *Caenorhabditis elegans *(Worm). The number of miRNAs were 83, 341 and 115 respectively. Only experimentally validated sequences as annotated by miRbase was used for the analysis. We selected three organisms to test the efficacy of the method on datasets derived from evolutionarily distinct species. While Chimpanzee and mouse are evolutionarily closer mammals, worms are evolutionarily distant and have been used extensively for comparative genomics studies. We have compared our method with all three *ab-initio *prediction methods for these organisms.

### Average Frequencies of sequence-structure feature elements

**Figure 2:**
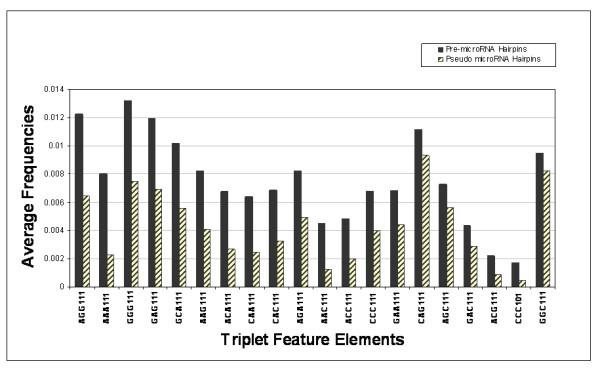
**Average frequencies of the top 20 differentiating feature elements in experimentally validated microRNA precursor hairpins in comparison to pseudo microRNA hairpins**.

**Figure 3:**
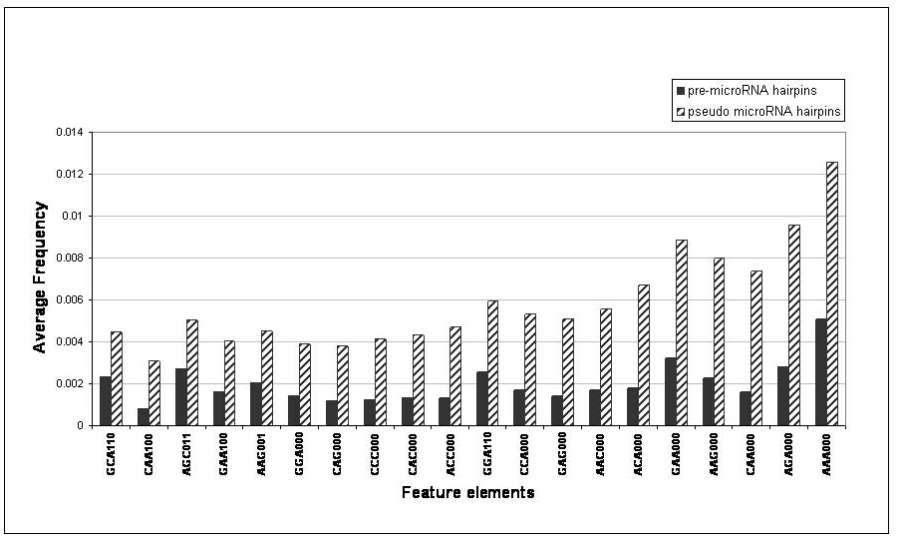
**Average frequencies of the top 20 differentiating feature elements in pseudo microRNA precursor hairpins in comparison to experimentally validated microRNA hairpins**.

Average frequencies of the feature elements were compared between the positive set (validated microRNA precursor hairpins) and the negative set (pseudo microRNA hairpins) to derive the frequently occurring and infrequently occurring sets of features. 20 most frequent and infrequent features are plotted in Fig 2 and 3.

The finding shown in Figure 2 that continuously paired triplet elements (NNN111) occur in high frequency in microRNA precursor hairpins than in pseudo microRNA hairpins and that continuously unpaired (NNN000) elements have a higher frequency in pseudo microRNA hairpins agrees with earlier analysis [23] of structural elements. This also reiterates the fact that microRNA precursor hairpins are well ordered structures in comparison to non-microRNA hairpins. This also agrees with the previous finding by Kim et al [25]

## Discussion

In the present study we demonstrate that incorporating sequence-structure triplet elements can be used for efficient *ab-initio *prediction of microRNA hairpins. Recently a number of *ab-initio *prediction methods for microRNAs have emerged, based on machine learning approaches. These include ProMiR [19], BayesmiRNAfind[24], TripletSVM [23] and mir-abela. ProMiR is one of the first machine learning based approaches proposed in the literature for the discovery of microRNAs. BayesmiRNAfind relies on Bayesian models while TripletSVM and mir-abela are based on Support Vector Machines (SVM). Triplet SVM takes into consideration a triplet structural element along with the nucleotide sequence in the mid-position of the triplet element. Thus although TripletSVM incorporates both sequence and structure the emphasis is laid on the structural feature. Similarly mir-abela, also uses SVM to classify microRNA precursor hairpins based on a host of structural features with a less emphasis on the sequence properties. On the other hand recent machine learning method, BayesmiRNAfind uses detailed structural and thermodynamics features with sequence elements for prediction and employs a Naïve Bayes classification.

In the method proposed here, we strike a balance between sequence and structure elements by using chimeric feature elements encompassing nucleotides and their base-pairing states in the structure. We use the RBF kernel of SVM to create a function corresponding to the hyper-surface that optimally separates true and pseudo microRNA hairpins.

Though our method performs better than other methods reviewed for mammalian as well as virus expressed microRNA precursors, the other methods outperform our method in predicting microRNAs expressed by *Caenorhabditis elegans *(Table 3). This may be partly due to the difference in sequence composition between human and *Caenorhabditis elegans*. The proposed approach can be further improved for prediction of eukaryotic microRNAs by training on a bigger dataset encompassing all experimentally validated eukaryotic microRNA hairpin sequences (manuscript in preparation). This method could also be modified to predict microRNAs expressed by viruses with a host range by training on host-specific datasets (for example, plants). Further improvements can be achieved by taking other parameters like thermodynamic features [26] and positional predisposition of particular nucleotides. Yet another way to increase accuracy of predictions is to club top performing algorithms for consensus predictions.

**Table 3:** Comparison of the prediction efficiency of TripletSVM and our method on eukaryotic microRNA hairpins derived from mirBase

**Experimentally validated microRNAs derived from miRbase**	**PREDICTIONS**			
**Virus**	**Bayes miRNAfind**	**Mir-abela**	**tripletSVM**	**MODEL 031**

*P troglodytes* **(83)**	72 **86.74**	68 **81.92**	71 **85.54**	73 **87.95**

*M musculus* **(341)**	272 **79.76**	227 **66.56**	276 **80.93**	285 **83.57**

*C elegans* **(115)**	76 **66.08**	78 **67.82**	86 **74.78**	74 **64.34**

The numbers denote the total number of positive predictions. The number in brackets following the organism name denotes the total number of entries in miRbase and that following the number of positive predictions is the percentage positive predictions.

In summary we describe a novel method based on machine learning which performs better than recently reported methods for *ab-initio *prediction of microRNA hairpins. The algorithm is fast and efficient and can scale for genome-scale predictions not only on viral genomes, but also on much larger eukaryotic genomes.

## Materials and methods

### MicroRNA Precursor Sequences

Human microRNA hairpin sequences were downloaded from miRbase [27](release 8.0) as on May 31, 2006 and contained 462 unique sequences. The dataset was manually curated to exclude sequences with no experimental validation (as annotated by miRbase). The final dataset comprised of 377 microRNA precursor sequences of varying lengths, with an average length of ~90 nucleotides.

The hairpin sequences for other eukaryotes were similarly derived from miRbase. The virus expressed microRNA hairpins for Epstein Barr virus (EBV), Cytomegalovirus (CMV), and Kaposi Sarcoma Herpes virus (KSHV) were also derived from miRbase. The two validated microRNA hairpin sequences from Herpes Simplex Virus Type I (HSV-1) were derived from the respective manuscripts [10,11].

### Pseudo-microRNA sequences

We created a set of sequences of length 90 nt derived from coding regions of genes with no alternate transcripts. The coding sequences were batch downloaded from Ensembl [28] (Ensembl 35) using the Martview feature. The sequence was chopped into non-overlapping 90mer fragments and checked for the propensity to form hairpin sequences. RNAfold program which is part of the freely available Vienna RNA Package [29], employing Zuker's algorithm was used for this purpose. The sequences which formed hairpins excluding internal loops and having a free energy of less than -15 Kcalmol-1 were filtered using in-house developed Perl scripts. A set of 430 such sequences were selected randomly from the entire set and used as the negative set for training.

### Processing of Datasets

Both the positive and negative sets were tagged and encoded into the native format for SVM with corresponding values for each of the 512 vectors (see details below) normalized to the total number of triplets possible for a particular sequence using in-house developed Perl scripts. RNAfold was used to determine the secondary structure of microRNA precursors.

### Training and test datasets

A set of about half the number of sequences in each dataset (positive and negative) was picked up randomly for training and the remainder formed the dataset for testing the model. Models were generated by creating one hundred such random samples and were evaluated simultaneously. The models were named by numbers which denoted the sample.

### Support Vector Machine

Support Vector Machine is a supervised machine learning method for generating functions for training data and is based on statistical and optimizing theory. In the Support Vector Machine algorithm, the datasets belonging to different classes are tagged, encoded as feature vectors and are mapped onto a feature space by the kernel function, with the aim of seeking a function that defines a global hyper-plane that would optimally separate the classes of training vectors. Support Vector Machine has been popular for quite some time due to its ability to handle large datasets and large feature spaces.

SVM was implemented using SVM^light^. SVM^light ^is an implementation of the Vapnik's Support Vector Machine algorithm [30] originally created for solving pattern recognition problems. The optimization algorithms used in SVM^light ^are described in [31]. The algorithm has scalable memory requirements and can handle problems with many thousands of support vectors efficiently.

The implementation enables the user to define a number of parameters as well as to select from a choice of inbuilt kernel functions. We employed RBF kernel and used a grid search strategy varying combinations of penalty parameter C and the Radial Bias Function (RBF) kernel parameter γ. The performance of each model was assessed based on specificity and sensitivity.

### Feature Vectors/Elements

Sequence structure features are known to play a critical role in microRNA processing [26]. We employed a feature space which encompasses sequence and its structural context at the same time. The sequence is folded using RNAfold and the structural context of overlapping triplets is determined. A triplet nucleotide can have 64 possibilities and each nucleotide in the triplet can have two states, 1 if it is bound and 0 if it is unbound. Thus such a chimeric feature (eg AUG001, AUG010 ... etc) can have a total of 512 (i.e., 4^3*2^3) possibilities.

The feature content is calculated using the formula



where (i) represents one of the 512 features.

### Web implementation

The method described in this paper is implemented as a web-based server [32]

## Abbreviations

SVM: Support Vector Machine; RISC: RNA Induced Silencing Complex; LAT: Latency associated transcript; EBV: Epstein Barr Virus; KSHV: Kaposi Sarcoma Herpesvirus; CMV: Cytomegalovirus

## Competing interests

The authors declare that they have no competing interests.

## Authors' contributions

VS formulated the idea. Data was collected by SK and resource was implemented by SK and FAA. All authors contributed to writing the manuscript.
